# Retraction: Reduction of WDR81 impairs autophagic clearance of aggregated proteins and cell viability in neurodegenerative phenotypes

**DOI:** 10.1371/journal.pgen.1009746

**Published:** 2021-08-17

**Authors:** Xuezhao Liu, Limin Yin, Tianyou Li, Lingxi Lin, Jie Zhang, Yang Li

Following publication of this article [1], the authors informed the *PLOS Genetics* Editors that results from experiments reported in Fig 5M and 5N were not reproducible in follow-up experiments, raising questions about the validity of the main conclusions.

In Fig 5M and 5N of this article [1], the authors report experiments using fibroblasts from two Huntington’s disease (HD) patients with two different polyQ expansions (Htt47Q, n = 1; Htt68Q, n = 1; control individuals, n = 2). These results are derived from three independent experiments. However, after the publication of this article [1], the authors collected more samples of fibroblasts from HD patients and control individuals for further experiments. It came to the authors’ attention that knocking-down of WDR81 did not aggravate cell death under proteotoxic stress in the fibroblasts from these additional samples from HD patients (Htt83Q and Htt92Q). Furthermore, overexpression of WDR81 did not restore the cell viability of fibroblasts from HD patients with different polyQ expansions, including Htt83Q and Htt92Q.

Given the results noted above, the authors consider the conclusions, and in particular the results reported in Fig 5M and 5N, invalid. Therefore, the authors are retracting this publication.

The authors apologize to the readers and editors of *PLOS Genetics* for the inconvenience.

All authors agreed with the retraction.
